# Dihydromyricetin ameliorates diet-induced obesity and promotes browning of white adipose tissue by upregulating IRF4/PGC-1α

**DOI:** 10.1186/s12986-022-00672-6

**Published:** 2022-06-11

**Authors:** Qingyang Leng, Jianhua Zhou, Chang Li, Yanhong Xu, Lu Liu, Yi Zhu, Ying Yang, Hongli Zhang, Xiaohua Li

**Affiliations:** 1 Department of Endocrinology, Seventh People’s Hospital of Shanghai University of Traditional Chinese Medicine, Shanghai, China; 2 Department of Endocrinology and Metabolism, Shanghai Clinical Center for Diabetes, Shanghai Key Clinical Center for Metabolic Disease, Shanghai Diabetes Institute, Shanghai Key Laboratory of Diabetes Mellitus, Shanghai Jiao Tong University Affiliated Sixth People’s Hospital, Shanghai, China

**Keywords:** Dihydromyricetin, Obesity, Browning of WAT, PGC-1α, IRF4

## Abstract

**Background:**

Promoting the browning of white adipose tissue (WAT) is a promising approach for the treatment of obesity and related comorbidities because it increases energy expenditure. In this study, we investigated whether Dihydromyricetin (DHM), a flavonoid component, could ameliorate diet-induced obesity through promoting the browning of WAT.

**Methods:**

Male C57BL/6 J mice were received a high-fat diet (HFD) to induce obesity and subsequently were treated with DHM (100 mg/kg/day) or vehicle for 4 weeks. The effects of DHM on weight reduction and metabolic phenotype improvement were observed in the mice. The expression of genes and protein involved in browning of WAT were assessed in inguinal WAT (iWAT) of the mice. Then, the effect of DHM on the inducing browning program was verified in adipocytes differentiated from stromal vascular fraction (SVF) cells of mouse iWAT. Finally, the mechanism by which DHM improves the browning of WAT was explored using RNA-seq and luciferase reporter assay.

**Results:**

We find that DHM reduces body weight, decreases WAT mass, improves glucose and lipid metabolic disorders, and ameliorates hepatic steatosis in diet-induced obese (DIO) mice. Further studies show that DHM induces WAT browning, which is manifested by increased expression of uncoupling protein 1 (UCP1) and peroxisome proliferator-activated receptor gamma coactivator (PGC)-1α and enhanced mitochondrial activity in iWAT and primary adipocytes. In addition, we also find that DHM enhances interferon regulatory factor 4 (IRF4) expression, which is a key transcriptional regulator of PGC-1α.

**Conclusion:**

Our findings identify that DHM prevents obesity by inducing the browning of WAT through the upregulation of IRF4/PGC-1α, which may have potential therapeutic implications for the treatment of obesity.

**Supplementary Information:**

The online version contains supplementary material available at 10.1186/s12986-022-00672-6.

## Background

In recent years, obesity has spread globally, similar to an epidemic. According to data published by the World Health Organization in 2016, there are currently more than 1.9 billion adults who are overweight worldwide, of whom more than 650 million people are obese [1]. Obesity is a major risk factor for metabolic disorders, such as type 2 diabetes, nonalcoholic fatty liver disease (NAFLD), hypertension and cardiovascular disease [2], and contributes to global disease and financial burden. Therefore, searching for novel effective treatments is a priority.

Obesity occurs when the body’s energy input continually exceeds its output. Adipose tissue is multifunctional and plays an important role in whole-body energy homeostasis [3, 4]. In mammals, three adipocyte subtypes with distinct functions have been identified. White adipocytes are mainly responsible for storing energy in the form of triglycerides (TGs) [5]. Brown adipocytes are mainly involved in energy consumption through thermogenesis. Adipocytes highly express UCP1, a protein located in the mitochondrial inner membrane that uncouples mitochondrial respiration from ATP synthesis, which can dissipate chemical energy as heat [6]. Beige adipocytes, also called “inducible brown adipocytes”, are interspersed in WAT; can express UCP1 as a result of various stimulations, such as chronic cold exposure and β3-adrenergic agonists; and can contribute to energy expenditure [7]. This phenomenon is often known as “browning” of WAT. Recently, the antiobesity effect of beige adipocytes has been widely confirmed [8–10]. Therefore, promoting WAT browning has become a hot topic in the field of obesity treatment and an important direction for the development of new antiobesity drugs.

Previous studies have shown that vine tea plays an important role in reducing Western diet-induced body weight and ameliorating lipid accumulation in the liver [11, 12]. DHM is the most bioactive flavonoid component in vine tea. It has been reported that DHM can exert anti-inflammatory, antioxidative stress and antitumor effects [13–15]. In addition, DHM improves NAFLD by increasing hepatocyte mitochondrial respiratory capacity and redox homeostasis [16] and increases insulin sensitivity by upregulating the tyrosine phosphorylation of insulin receptor substrate 1 (Y612) in db/db mice [17]. Further studies have shown that DHM has an antiadipogenic effect in adipocytes by reducing peroxisome proliferator-activated receptor γ (PPARγ) phosphorylation at serine 273 and directly interacting with 78-kDa glucose-regulated protein [18, 19]. The anti-obesity effect of DHM by promoting WAT browning is less studied.

In the present study, we find that DHM can reduce body weight and improve glucose and lipid metabolic disorders in DIO mice. Moreover, DHM also stimulates beige formation in iWAT and primary adipocytes and increases energy expenditure by activating the IRF4/PGC-1α signaling pathway.

## Materials and methods

### Mice

Male C57BL/6 J mice (6–8 weeks old) were purchased from Shanghai SLAC Laboratory Animal Company and received a HFD with 60% of kcal from saturated fat (Research Diets, New Brunswick, NJ) for 10–12 weeks. Then, the mice were randomly subdivided into two body weight-matched groups: a HFD + DHM group, which was treated with DHM (100 mg/kg/day) daily by gavage, and a HFD + vehicle group, which was administered the same volume of vehicle (normal saline) daily by gavage. Food intake and body weight were recorded weekly. After 4 weeks of treatment, the mice were killed, and the adipose tissues and liver were separated and weighed. The bean-size tissues were fixed in 4% paraformaldehyde, and the rest of the tissues were stored at -80 °C.

### Metabolic phenotype analyses

An intraperitoneal glucose tolerance test (IPGTT) and an in vivo insulin tolerance test (ITT) were performed 4 weeks after DHM treatment. In the IPGTT, mice were injected intraperitoneally with 2 g dextrose/kg body weight after an overnight fast. Blood glucose concentrations were measured at various time points (0, 15, 30, 60, and 120 min). For ITT, the mice were fasted for 6 h. The blood glucose concentrations were measured at 0, 15, 30, 45 and 60 min after intraperitoneal insulin injection (0.75 IU/kg). Tail vein blood glucose was determined by using an Accu-Chek Performa glucometer (Roche, Basel, Switzerland).

TGs and total cholesterol (TC) were measured by an automatic biochemical analyzer (Shanghai Fenglin Medical Laboratory Co., Ltd, Shanghai, China).

### Histologic analysis and immunohistochemistry

Tissues fixed in 4% paraformaldehyde were embedded and cut into slices. Then, the slices were stained with hematoxylin and eosin (H&E) for morphological analysis. For UCP1 staining, the slices were deparaffinized, hydrated, incubated with hydrogen peroxide blocking solution, boiled in antigen repair solution (1 mM Tris–EDTA, pH 9.0) for 15 min, blocked with goat serum for 20 min at 37 °C, incubated with anti-UCP1 antibody overnight at 4 °C and then incubated with the secondary antibody for 30 min at 37 °C.

### SVF cell isolation and beige adipocyte differentiation

SVF cells obtained from iWAT of male C57BL/6 J mice at 6–8 weeks old as previously described [20] were cultured in 10% FBS-DMEM. Confluent cells were cultivated in medium containing 10% FBS, 5 mg/ml insulin (Lily), 0.5 mM isobutyl methylxanthine (Sigma), 1 mM dexamethasone (Sigma), 1 μM rosiglitazone (Sigma) and 1 nM T3 (Sigma). After 2 days, the cells were switched to medium with insulin and T3 for 4 days.

### Oil red O staining

Mature beige adipocytes that were treated with 5 μM DHM for 24 h were washed twice with phosphate-buffered saline (PBS), fixed in 4% paraformaldehyde for 5 min, and stained with oil red O working solution for 15 min. Finally, the stained cells were imaged with a microscope (Nikon Corp, Japan) after being washed with PBS.

### Cell oxygen consumption rate (OCR) measurements

SVF cells were inoculated in XF24 cell culture microplates coated with poly-L-lysine. On the 4th day of cell differentiation into beige adipocytes, the cells were administered 5 μM DHM for 24 h. Then, the cell OCR was determined as previously described [21] by an XF24 analyzer (Seahorse Bioscience, USA). Finally, the OCR results were normalized to the protein concentration.

### Plasmid transfection and luciferase reporter assay

HEK293T cells were transfected with PGC-1α or IRF4 promoter luciferase reporter plasmids using Lipofectamine 3000 (Invitrogen). The PGC-1α promoter plasmid was a gift from Professor Zhiguo Zhang. IRF4 promoter luciferase reporter constructs were generated based on research by Jun Eguchi [22]. Twenty-four hours after transfection, the cells were treated with 5 μM DHM for 24 h. Then, the cells were collected, and the luciferase activity was assessed with the dual-luciferase reporter assay system (Promega).

### Western blot analysis and antibodies

Total protein from tissues or cells was prepared with RIPA lysis buffer supplemented with protease and phosphatase inhibitors (Roche). The protein samples were boiled for 10 min and then were separated by 10% sodium dodecyl-sulfate polyacrylamide gel electrophoresis (SDS-PAGE) and transferred to nitrocellulose membranes (Millipore). Next, the membranes were blocked with 5% milk for 1 h at room temperature and incubated with the corresponding antibodies against UCP1 (Abcam), PGC-1a (Millipore), β-Actin (Cell Signaling Technology), AMPK (Cell Signaling Technology),Phospho-AMPKα (Thr172) (Cell Signaling Technology), p38 MAPK (D13E1)XP® (Cell Signaling Technology),Phospho-p38 MAPK (Thr180/Tyr182) (Cell Signaling Technology), Phospho- (Ser/Thr) PKA Substrate (Cell Signaling Technology), Tubulin (Sigma) and IRF4 (Santa Cruz) overnight at 4 °C. Subsequently, the membranes were incubated with the secondary antibody for 1 h at room temperature. An ECL detection system was used to detect the signals (GE Healthcare, USA).

### Real-time quantitative PCR analysis (RT-qPCR)

Total RNA was extracted from tissues or cells with TRIzol reagent (Invitrogen). RNA (1 µg) was reverse-transcribed into cDNA with a Prime Script® RT reagent Kit (Takara). RT-qPCR analysis was performed with SYBR Premix Ex Taq (Takara) in a LightCycler 480 PCR system (Roche, Germany). The housekeeping gene 36B4 was used to normalize the quantitative expression of targeted genes, which were counted using the 2 − ΔΔCT method. All primer sequences are collected in Additional file 1: Table S1.

### Statistical analysis

SPSS 20 statistical software was used to analyze the data, and the data are expressed as the mean ± SEM. Student’s t-test was used to compare the two groups, where *, **, and *** represent *P* < 0.05, *P* < 0.01, and *P* < 0.001, respectively, compared with the control group.

## Results

### DHM ameliorates obesity in DIO mice

**Fig. 1:**
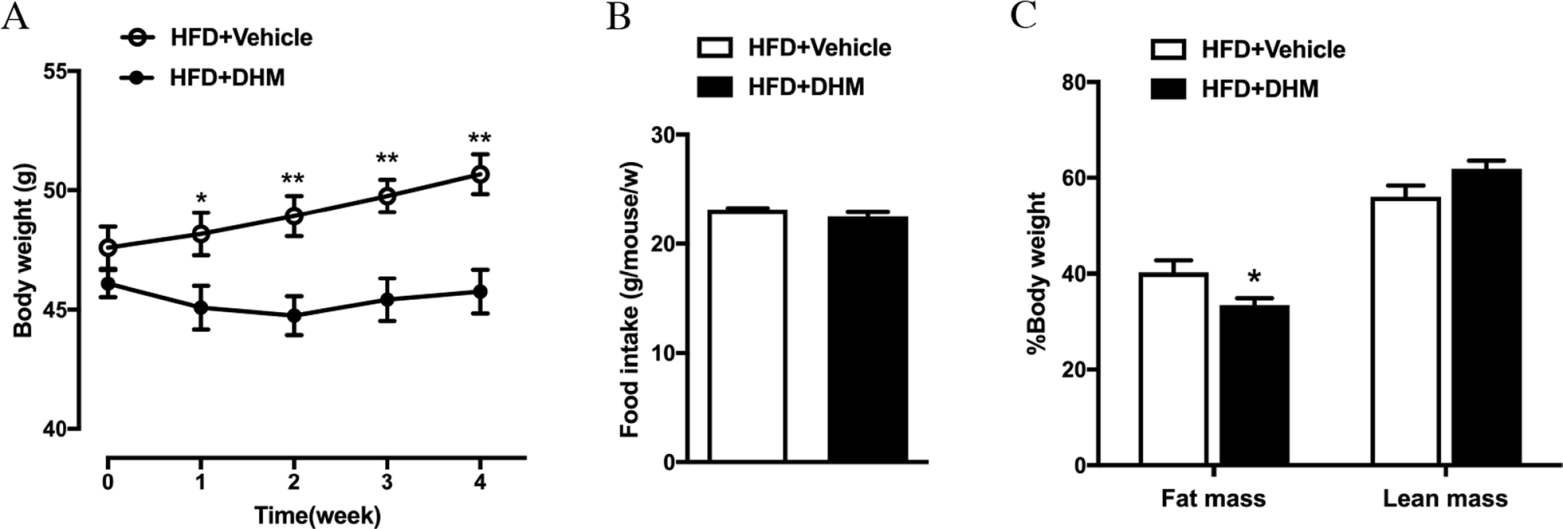
DHM prevents diet-induced obesity. **A** Body weight of DIO mice during DHM administration (n = 6). **B** Food intake per week during the 4 weeks of the experiment (n = 8). **C** Fat mass and lean mass normalized by body weight (n = 5). Data are presented as the mean ± SEM. *P < 0.05, **P < 0.01

To investigate the effect of DHM on body weight, DIO mice were orally administered vehicle or DHM for 4 weeks. There was no significant difference in body weight between the groups before drug administration. After one week of administration, the body weight of mice treated with DHM began to be reduced compared to that of the mice administered vehicle, and the difference between the groups increased gradually with prolonged administration time (Fig. 1A). However, there was no significant change in food intake between the groups (Fig. 1B), suggesting that DHM does not change energy intake. In addition, the body composition of the mice was detected by an NMR body composition analyzer for small animals. The fat mass of the DHM-treated mice was significantly reduced compared with that of the vehicle-treated mice, while there was no change in lean mass between the groups (Fig. 1C), suggesting that DHM can reduce body weight by decreasing the fat content of DIO mice.

### DHM prevents metabolic dysfunction in DIO mice

**Fig. 2:**
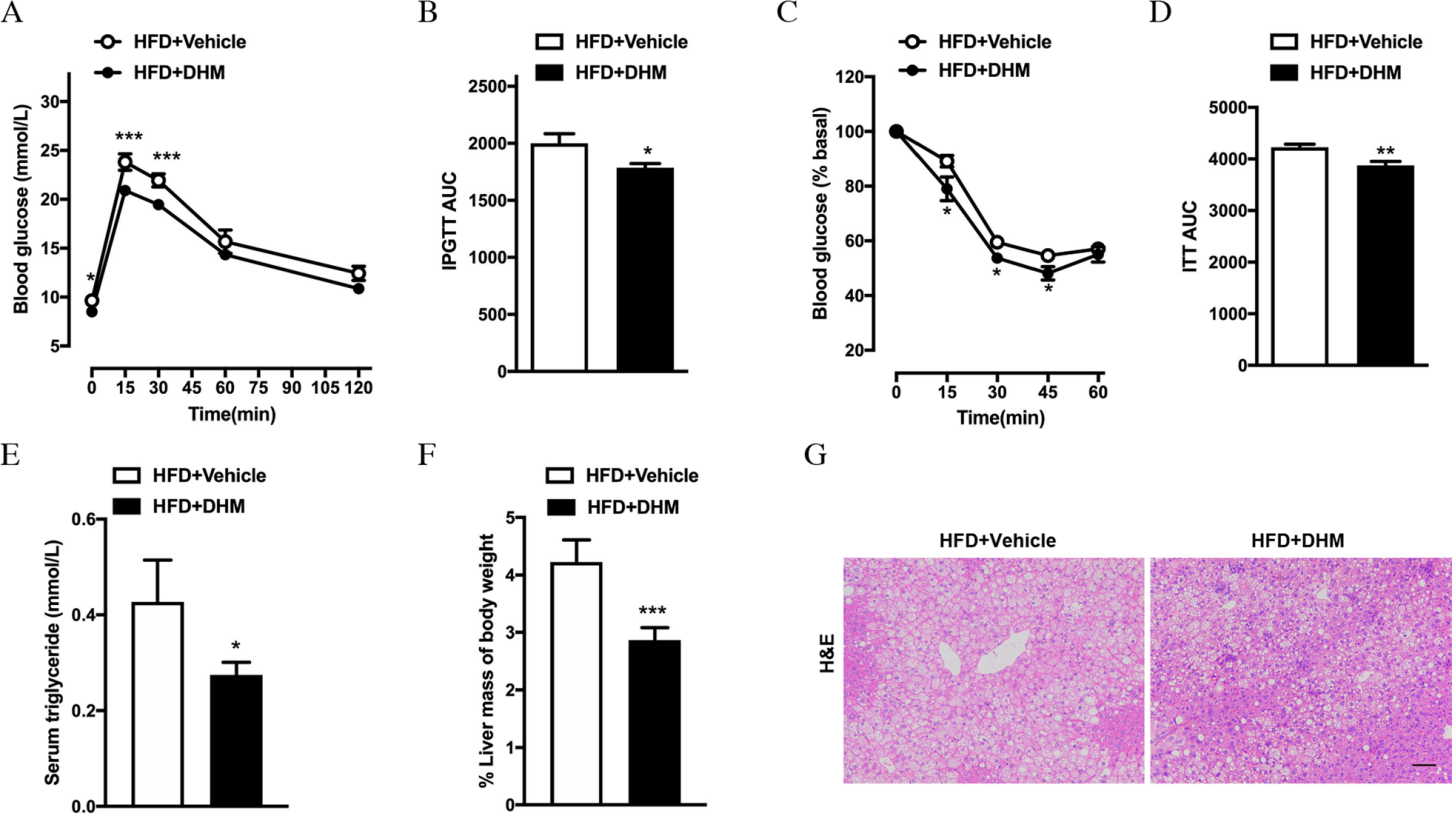
DHM protects against metabolic dysfunction in DIO mice. **A**, **B** Intraperitoneal glucose tolerance test (n = 8). **C**, **D** Insulin tolerance test (n = 8). **E** Serum triglyceride level (n = 5). **F** Liver mass normalized by body weight (n = 5). **G** Representative liver H&E staining(n = 5). Scale bar = 100 μm. Data are presented as the mean ± SEM. *P < 0.05, **P < 0.01, and ***P < 0.001

Obesity is often accompanied by metabolic disorders of glucose and lipids. Therefore, we wanted to know whether the DHM-induced reduction in fat mass can improve the glucose and lipid profile of DIO mice. After 4 weeks of drug treatment, the IPGTT was carried out. The results showed that the blood glucose level at 0, 15, and 30 min after intraperitoneal glucose injection and the area under the curve (AUC) were markedly reduced in DHM-administered mice compared with vehicle-treated mice (Fig. 2A, B). Moreover, the ITT results suggested that DHM can improve the insulin sensitivity of DIO mice (Fig. 2C, D). In addition, DHM significantly decreased the level of serum TGs in DIO mice (Fig. 2E) but had no effect on serum TC (Additional file 2: Fig. S1). Simultaneously, DHM also ameliorated hepatic steatosis. The DHM-treated mice showed a significant loss of liver mass (Fig. 2F). H&E staining revealed a dramatic reduction in lipid accumulation within the livers of DHM-treated mice (Fig. 2G). In conclusion, these results suggest that DHM can improve the metabolic profile.

### DHM promotes browning of iWAT

**Fig. 3:**
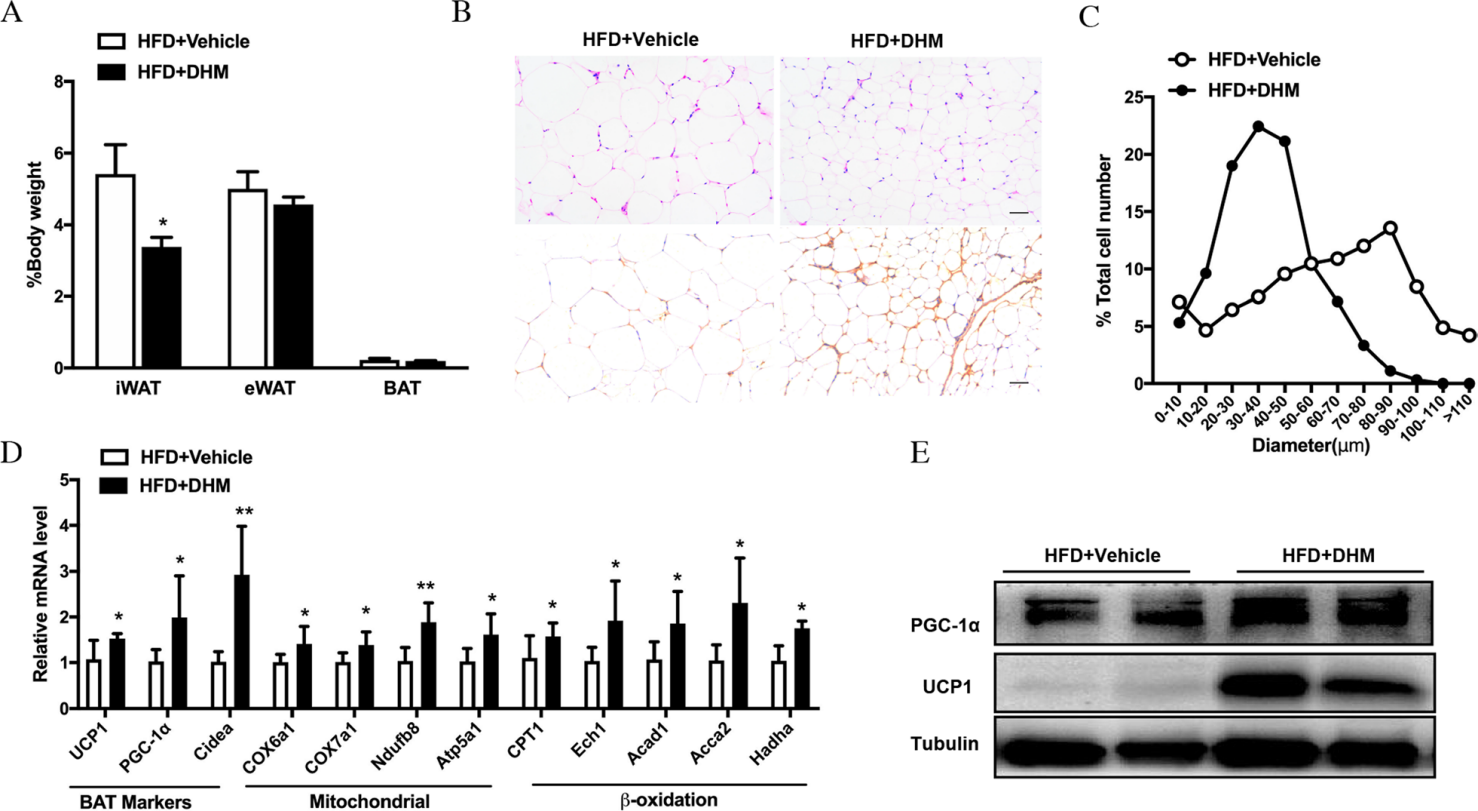
DHM stimulates iWAT browning. **A** iWAT mass, epididymal WAT (eWAT) mass and BAT mass normalized by body weight (n = 5). **B** Representative H&E staining of iWAT (upper panel) and UCP1 immunohistochemistry (brown) (lower panel), scale bar = 100 μm. **C** Percentage of different adipocyte cell sizes in iWAT(n = 5). **D** RNA expression profiles of the brown fat marker and mitochondrial-related and fatty acid oxidation-related genes in iWAT. **E** PGC-1α and UCP1 protein expression in iWAT. Data are presented as the mean ± SEM. *P < 0.05, **P < 0.01

In accordance with the reduction in fat mass, the DHM-treated mice had less iWAT mass than the mass of the control mice (Fig. 3A). The H&E staining data revealed that the size of adipocytes in the iWAT of mice treated with DHM was smaller (Fig. 3B-C). Moreover, there were some multilocular adipocytes in the iWAT, which is a morphological characteristic of brown adipose tissue (BAT) (Fig. 3B). Subsequently, immunohistochemical staining of UCP1 was performed. The results showed that UCP1 staining was significantly elevated after DHM administration (Fig. 3B). Moreover, we observed that DHM not only markedly upregulated the mRNA expression levels of brown fat marker genes such as UCP1 and PGC-1α but also increased the expression of mitochondrial-related genes and fatty acid oxidation-related genes (Fig. 3D). However, there were no significant changes of mRNA expression levels of adipocyte differentiation and lipogenesis genes such as Fabp4, PPARγ, Glut4, Adipoq (Additional file 2: Fig. S2) in the two groups. In agreement, the protein levels of UCP1 and PGC-1α were also enhanced in the DHM group (Fig. 3E). The results indicated that DHM may ameliorate diet-induced obesity by inducing browning of iWAT without inhibiting adipocyte differentiation and lipid synthesis.

### DHM stimulates the browning of primary adipocytes and increases energy expenditure

**Fig. 4:**
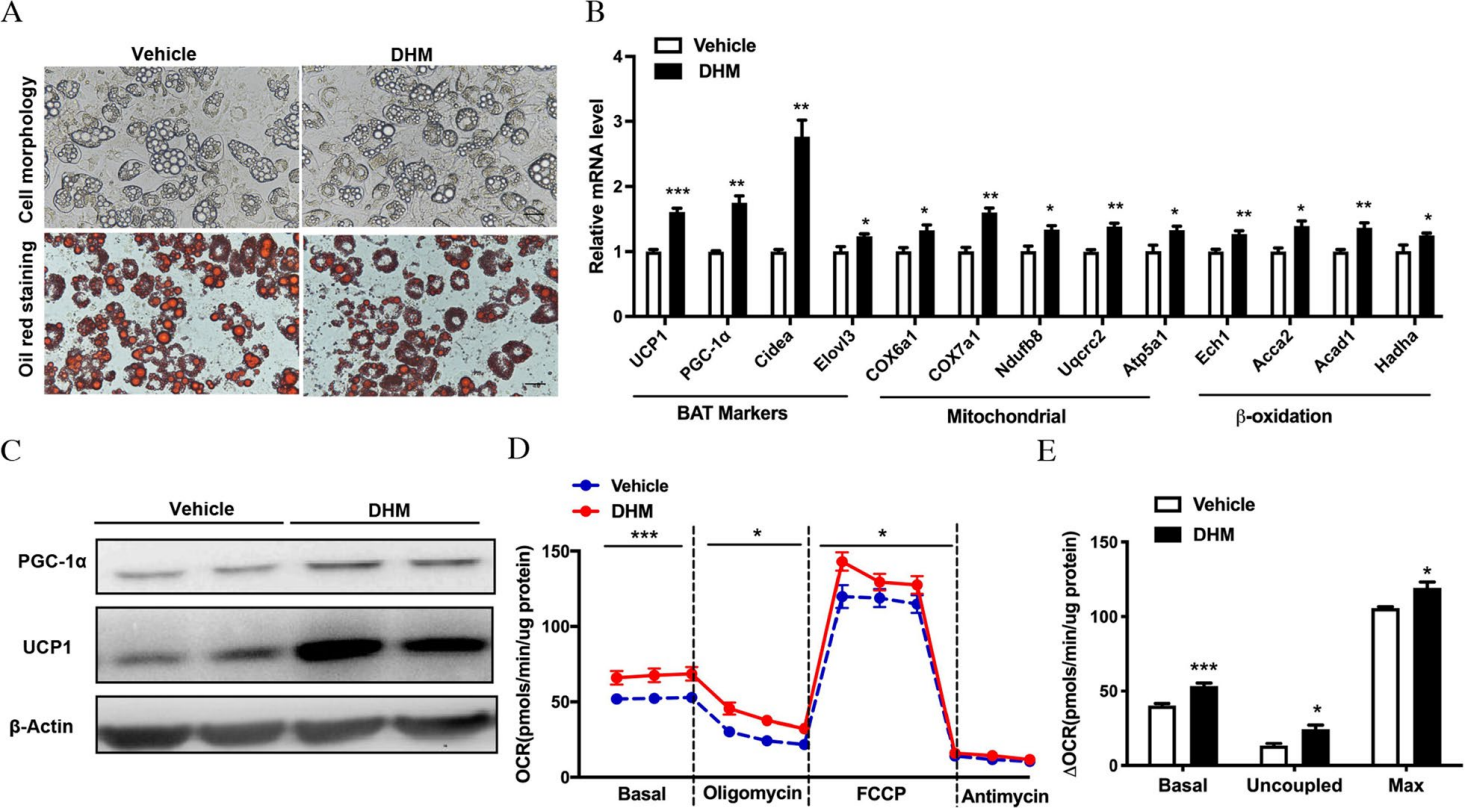
DHM induces the browning of primary adipocytes and increases mitochondrial activity. **A** Representative images of visual estimation and oil red O staining of primary inguinal adipocytes treated with DHM, scale bar = 100 μm. **B** RNA expression profiles of the brown fat marker and mitochondrial-related and fatty acid oxidation-related genes in primary inguinal adipocytes. **C** Western blots of PGC-1α and UCP1 protein in primary inguinal adipocytes. **D**, **E** Basal OCR in DHM-treated primary inguinal adipocytes. Data are presented as the mean ± SEM. *P < 0.05, **P < 0.01, and ***P < 0.001

To investigate whether iWAT browning was mediated by a direct effect, adipocytes differentiated from SVF cells of mouse iWAT were treated with DHM (5 µM). As expected, visual estimation and oil red O staining by phase contrast microscopy showed that DHM-treated adipocytes had smaller lipid droplets than those of control cells (Fig. 4A). In addition, the UCP1 and PGC-1α mRNA and protein expression levels were markedly enhanced (Fig. 4B-C). Similarly, DHM increased the mRNA expression levels of mitochondrial-related genes and fatty acid oxidation-related genes (Fig. 4B), but did not alter adipocyte differentiation and lipogenesis genes (Additional file 4: Fig. S3), indicating that DHM had no effect on adipocyte differentiation and lipid synthesis.The results suggested that DHM could stimulate the browning of primary adipocytes. To reveal the impact of DHM on mitochondrial activities, a mitochondrial stress test was carried out, and the results showed that basal oxygen consumption, ATP synthesis, oxygen consumption and maximum oxygen consumption were significantly elevated in primary adipocytes after treatment with DHM (Fig. 4D-E), indicating that DHM could increase the energy consumption of mature adipocytes.

### DHM improves the browning of WAT by upregulating IRF4/PGC-1α

**Fig. 5:**
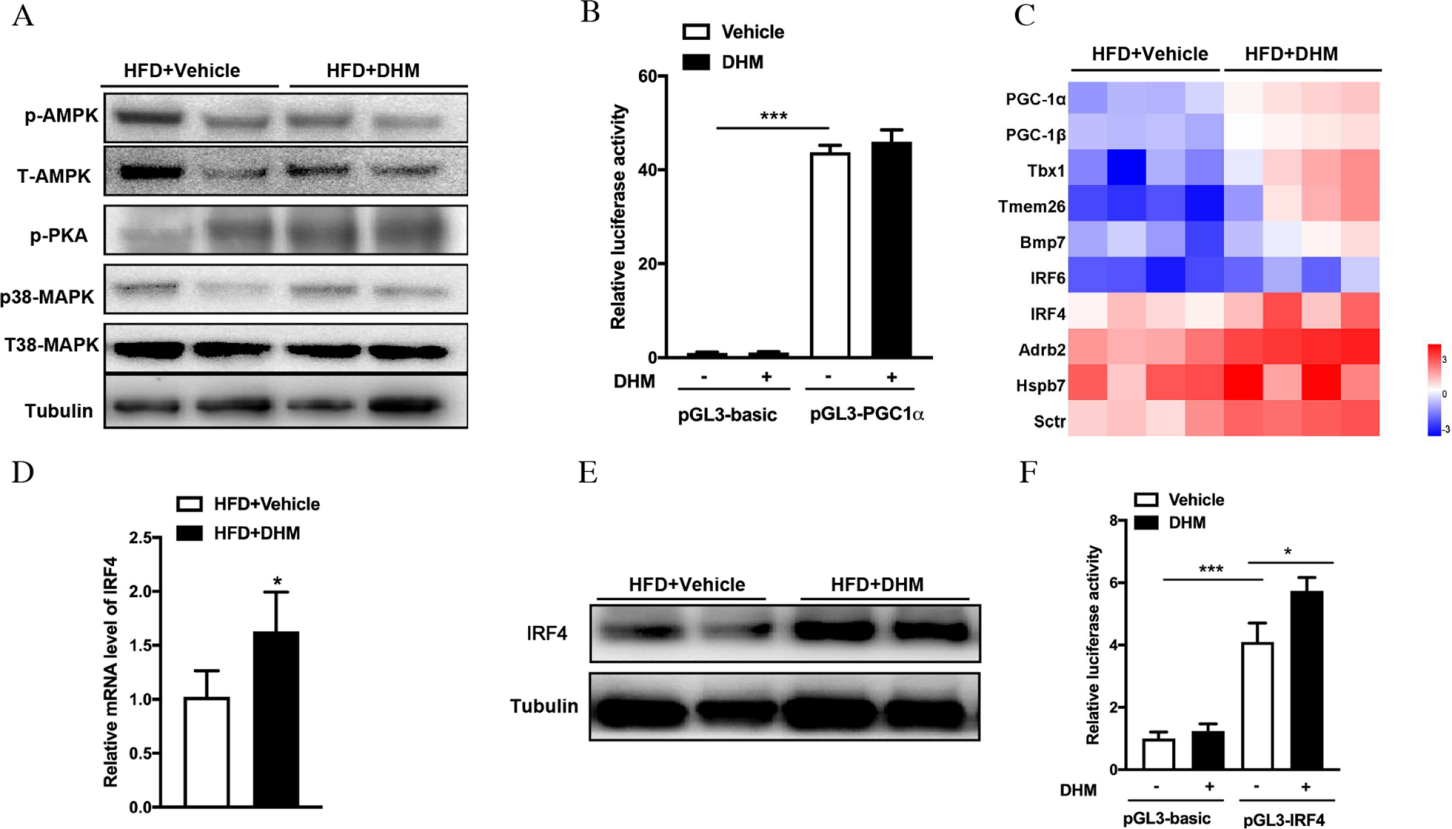
DHM induces the browning program by upregulating IRF4/PGC-1α. **A** Protein levels of p-AMPK, T-AMPK, p-PKA, p-p38 MAPK, and T-p38 MAPK in iWAT. **B** Luciferase expression of the PGC-1α promoter in HEK293T cells. **C** Heatmaps representing the expression of thermogenic genes in iWAT by RNA-seq. **D** The IRF4 mRNA expression level in iWAT. (E) The IRF4 protein expression level in iWAT. **F** Luciferase expression of the IRF4 promoter in HEK293T cells. Data are presented as the mean ± SEM. *P < 0.05, **P < 0.01, and ***P < 0.001

Our studies and others showed that DHM could upregulate the expression of PGC-1α, a crucial transcriptional coactivator, which could promote UCP1 expression  in  WAT [23, 24]. To illuminate the potential mechanism of the DHM-induced PGC-1α increase in adipocytes, the expression levels of critical signaling molecules involved in regulating PGC-1α expression, such as Adenosine 5 ‘-monophosphate activated protein kinase (AMPK) and p38 Mitogen-activated protein kinase (MAPK), were determined. However, our results showed that there were no significant changes in the above two signaling pathways after treatment with DHM (Fig. 5A). To further investigate whether DHM directly induced PGC-1α transcription, we performed a dual-luciferase reporter gene assay. The results showed that DHM did not increase the activity of the PGC-1α promoter (Fig. 5B), suggesting that DHM could not directly promote the transcription of PGC-1α. Subsequently, transcriptome sequencing was performed to illuminate the potential mechanism of DHM-induced PGC-1α expression. The results revealed that in addition to increasing the expression of PGC-1α, DHM could also enhance IRF4 expression (Fig. 5C). IRF4 has previously been reported to promote the expression of PGC-1α and to physically interact with PGC-1α to stimulate UCP1 expression [25]. As expected, the IRF4 mRNA and protein expression levels were markedly increased in the iWAT of DHM-treated mice (Fig. 5D-E). Moreover, DHM increased the luciferase expression of the IRF4 promoter (Fig. 5F), suggesting that DHM could directly promote the transcription of IRF4. Therefore, we infer that DHM promotes the browning of WAT by activating the IRF4/PGC-1α pathway.

## Discussion

A great deal of evidence suggests that converting white energy-storing adipocytes into brown-like energy-dissipating adipocytes represents an attractive tactic for protecting against obesity [26, 27]. Many studies have shown that small molecule compounds extracted from traditional Chinese medicines, such as berberine, cordycepin, and celastrol, can induce white adipose browning, increase heat production and reduce body weight [8, 9, 28]. Therefore, searching for more effective compounds that can promote browning programs is a priority. DHM has been reported to decrease blood glucose and blood lipid levels [16, 29, 30]. In the current study, our data showed that DHM had the capacity to decrease body weight, improve metabolic disorders of glucose and lipids and increase the consumption of energy by inducing WAT browning and offered new insight into the therapeutic action of this small molecule compound in obesity.

UCP1 is the hallmark protein of BAT. In addition, it can also be induced in inguinal WAT by chronic cold exposure and β3-adrenergic agonists and is capable of protecting against obesity and regulating energy homeostasis. UCP1 expression is mainly regulated by a variety of transcription factors, such as PPARγ, activating transcription factor 2 (ATF2), thyroid hormone receptor (TR), and PGC-1α [24]. The mechanism by which DHM upregulates UCP1 expression in beige adipocytes has not yet been reported, but studies have shown that the expression of PGC-1α in rat skeletal muscle cells is significantly upregulated by DHM [31]. It has been confirmed that PGC-1α is an important regulator that controls the expression of UCP1 in brown and beige adipocytes, which further reduces the proton gradient and uncouples oxidative phosphorylation, thereby enhancing energy consumption [32]. Furthermore, PGC-1α also plays a key role in regulating mitochondrial biogenesis and promoting thermogenesis. Upregulation of PGC-1α can trigger nuclear respiratory factor (NRF)-1 and NRF-2 activation; initiate the transcription of the mitochondrial genes β-ATP synthase, mitochondrial transcription factor A and cytochrome; and regulate the expression of oxidized respiratory chain subunits and the replication and transcription of mitochondrial DNA (mtDNA), thereby regulating fatty acid oxidation and mitochondrial biosynthesis [33]. As expected, our research showed that DHM could enhance PGC-1α expression in iWAT and increase the mitochondrial oxygen consumption of primary adipocytes, suggesting that DHM may promote the browning program by upregulating PGC-1α expression.

PGC-1α is an important transcriptional coactivator that regulates the body’s adaptive heat production, mitochondrial biosynthesis, and glucose and lipid metabolism [34]. The regulatory mechanism of PGC-1α expression is very complicated. PGC-1α is strictly regulated by many signal transduction effectors, such as AMPK, p38 MAPK, sirtuin-1 (SIRT1), and mammalian target of rapamycin (mTOR), which coordinate posttranslational modifications, such as phosphorylation and ubiquitination, to increase PGC-1α activity [35–37]. On the other hand, some transcription factors, such as the heat shock factor 1 (HSF1) and cAMP-response element binding protein (CREB), can directly or indirectly enhance PGC-1α transcription to increase its mRNA expression. At the same time, PGC-1α can also promote its own transcription through positive feedback [8, 33, 38]. Previous studies showed that small molecule compounds such as berberine and irisin mainly upregulated the expression of PGC-1α and promoted WAT browning by activating the AMPK and p38 MAPK signaling pathways. Given that the AMPK and p38 MAPK signaling pathways are vital for WAT browning, we next assessed whether DHM treatment had any effect on AMPK and p38 MAPK activity in iWAT. Our data showed that the AMPK and p38 MAPK activities were not significantly changed after DHM treatment, implying that DHM does not upregulate PGC-1α expression by activating the AMPK and p38 MAPK signaling pathways, although a previous study showed that DHM had an effect on activating the AMPK signaling pathway [39, 40]. The difference between our and others’ results may reflect the different effects of DHM in different tissues. Moreover, the results of a dual-luciferase reporter gene assay showed that DHM did not increase luciferase expression of the PGC-1α promoter, suggesting that DHM could not directly promote the transcription of PGC-1α. Fortunately, transcriptome sequencing showed that in addition to increasing the expression of PGC-1α, DHM could also enhance IRF4 expression. IRF4 is a transcription factor that is known for its function in regulating the immune system and oncogenesis [41]. Moreover, IRF4 participates in almost all metabolic activities of WAT, including inhibiting insulin-induced lipogenesis by downregulating the expression of genes related to adipogenesis, promoting lipolysis by upregulating the expression of adipocyte triglyceride lipase and hormone-sensitive lipase [22] and improving inflammation by promoting adipose tissue M2 macrophage polarization [42]. In addition, IRF4 is also a major transcriptional effector of thermogenesis that can promote PGC-1α expression and interact with PGC-1α to activate UCP1, thereby increasing energy expenditure [25]. Fortunately, our data showed that the IRF4 mRNA and protein expression levels were markedly enhanced after treatment with DHM, suggesting that DHM may promote the expression of PGC-1α by upregulating IRF4.

It has been reported that cold-induced IRF4 is simultaneously increased with PGC-1α and is necessary for full expression of PGC-1α. In the absence of IRF4, the expression of thermogenic genes, such as UCP1 and PGC-1α, cannot be induced, even in the case of PGC-1α overexpression. Furthermore, IRF4 expression was significantly decreased in the absence of PGC-1α. In summary, PGC-1α and IRF4 are mutually induced in a reciprocally reinforcing cycle. Moreover, there are strong physical interactions between IRF4 and PGC-1α. Previous studies have shown that there was an interferon-stimulated response element (ISRE) 1251 base pairs upstream of the UCP1 transcription start site. The UCP1 promoter can be driven independently by IRF4 and by PGC-1α, and the two together produce additive induction [22, 25]. Our data showed that the IRF4 promoter, rather than the PGC-1α promoter, can be driven directly by DHM, suggesting that DHM may induce the expression of IRF4; this IRF4 can promote the expression of PGC-1α and physically interact with PGC-1α to increase UCP1 expression, thereby playing a role in improving obesity. Of course, whether DHM-induced IRF4 expression promotes WAT browning through the above pathway still needs to be confirmed by further experiments. Moreover, how DHM transcriptionally regulates IRF4 expression is still unclear, and further research is still needed.

## Conclusions

In summary, our results demonstrate that DHM could promote the browning of WAT to offer a robust defense against obesity. We prove the mechanism by which DHM potently regulates the transcription of UCP1 in WAT through, at least in part, IRF4 and PGC-1α induction. These findings establish an important role for DHM in improving obesity and associated diseases.

## Supplementary Information

**Additional file 1. Table S1.** Primer Sequences Used in RT-qPCR

**Additional file 2. Fig. S1.** DHM had no effect on serum TC. Serum triglyceride level (n=5). Data are presented as the mean ± SEM. *P < 0.05, **P < 0.01, and ***P <0.001.

**Additional file 3. Fig. S2.** DHM did not inhibit lipid synthesis of iWAT. RNA expression profiles of the adipocyte differentiation and lipogenesis related genes in iWAT. Data are presented as the mean ± SEM. *P < 0.05, **P < 0.01, and ***P <0.001.

**Additional file 4. Fig. S3.** DHM had no effect on lipid synthesis of primary adipocytes. RNA expression profiles of the adipocyte differentiation and lipogenesis genes in primary adipocytes. Data are presented as the mean ± SEM. *P < 0.05, **P < 0.01, and ***P <0.001.

## Data Availability

All data of present study are available from the corresponding author on reasonable request.

## Abbreviations

WAT: White adipose tissue;
DHM: Dihydromyricetin
DIO: Diet-induced obese
UCP1: Uncoupling protein 1
PGC: Peroxisome proliferator-activated receptor gamma coactivator
SVF: Stromal vascular fraction
IRF4: Interferon regulatory factor 4
NAFLD: Nonalcoholic fatty liver disease
TGs: Triglycerides
PPAR γ: Peroxisome proliferator-activated receptor γ
HFD: High-fat diet
IPGTT: Intraperitoneal glucose tolerance test
ITT: Insulin tolerance test
TC: Total cholesterol
H&E: Hematoxylin and eosin
PBS: Phosphate-buffered saline
OCR: Cell oxygen consumption rate
SDS-PAGE: Sodium dodecyl-sulfate polyacrylamide gel electrophoresis
RT-qPCR: Real-time quantitative PCR analysis
AMPK: Adenosine 5 ‘-monophosphate activated protein kinase
MAPK: Mitogen-activated protein kinase
AUC: Area under the curve
BAT: Brown adipose tissue
ATF2: Activating transcription factor 2
TR: Thyroid hormone receptor
NRF: Nuclear respiratory factor
mt DNA: Mitochondrial DNA
SIRT1: Sirtuin-1
mTOR: Mammalian target of rapamycin
HSF1: Heat shock factor 1
CREB: CAMP-response element binding protein
ISRE: Interferon-stimulated response element
