# Supplementary material for: Dihydromyricetin ameliorates diet-induced obesity and promotes browning of white adipose tissue by upregulating IRF4/PGC-1α
Source: Nutr Metab (Lond). 2022 Jun 11;19:38. doi: 10.1186/s12986-022-00672-6 (PMC9188085; doi:10.1186/s12986-022-00672-6)
Supplement: Supplementary file 1 — Additional file 1. Table S1. Primer Sequences Used in RT-qPCR [file 12986_2022_672_MOESM1_ESM.docx]

Supplementary Material

**Supplementary Table 1.** Primer Sequences Used in RT-qPCR

| **Gene** | **Forward** | **Reverse** |
| --- | --- | --- |
| UCP1 | AGGCTTCCAGTACCATTAGGT | CTGAGTGAGGCAAAGCTGATTT |
| PGC-1α | AGCCGTGACCACTGACAACGAG | GCTGCATGGTTCTGAGTGCTAAG |
| Cidea | TGACATTCATGGGATTGCAGAC | CATGGTTTGAAACTCGAAAAGGG |
| COX6a1 | TCAACGTGTTCCTCAAGTCGC | AGGGTATGGTTACCGTCTCCC |
| COX7a1 | CAGCGTCATGGTCAGTCTGT | AGAAAACCGTGTGGCAGAGA |
| Ndufb8 | TGTTGCCGGGGTCATATCCTA | AGCATCGGGTAGTCGCCATA |
| Atp5a1 | TCTCCATGCCTCTAACACTCG | CCAGGTCAACAGACGTGTCAG |
| Elovl3 | TTCTCACGCGGGTTAAAAATGG | GGCCAACAACGATGAGCAAC |
| Uqcrc2 | AAAGTTGCCCCGAAGGTTAAA | GAGCATAGTTTTCCAGAGAAGCA |
| Ech1 | GCTACCGCGATGACAGTTTC | TCAGAGATCGAAGGCTGATGTT |
| Acaa2 | CTGCTACGAGGTGTGTTCATC | AGCTCTGCATGACATTGCCC |
| Acadl | TCTTTTCCTCGGAGCATGACA | GACCTCTCTACTCACTTCTCCAG |
| Hadha | TGCATTTGCCGCAGCTTTAC | GTTGGCCCAGATTTCGTTCA |
| CPT1 | GCACACCAGGCAGTAGCTTT | CAGGAGTTGATTCCAGACAGGTA |
| Adipoq | TGTTCCTCTTAATCCTGCCCA | CCAACCTGCACAAGTTCCCTT |
| Glut4 | GTGACTGGAACACTGGTCCTA | CCAGCCACGTTGCATTGTAG |
| PPARγ | TCGCTGATGCACTGCCTATG | GAGAGGTCCACAGAGCTGATT |
| Fabp4 | TTCGATGAAATCACCGCAGA | AGGGCCCCGCCATCT |
| IRF4 | GCAGCTCACTTTGGATGACA | CCAAACGTCACAGGACATTG |
| 36B4 | AAGCGCGTCCTGGCATTGTCT | CCGCAGGGGCAGCAGTGGT |

**Supplementary Figure**

**Figure legends**

**Fig. S1** DHM had no effect on serum TC.

Serum triglyceride level (n=5). Data are presented as the mean ± SEM. **P* < 0.05, ***P* < 0.01, and ****P* <0.001.

**Fig. S2** DHM did not inhibit lipid synthesis of iWAT.

RNA expression profiles of the adipocyte differentiation and lipogenesis related genes in iWAT. Data are presented as the mean ± SEM. **P* < 0.05, ***P* < 0.01, and ****P* <0.001.

**Fig. S3** DHM had no effect on lipid synthesis of primary adipocytes.

RNA expression profiles of the adipocyte differentiation and lipogenesis genes in primary adipocytes. Data are presented as the mean ± SEM. **P* < 0.05, ***P* < 0.01, and ****P* <0.001.
