# Supplementary figures and images for: Dihydromyricetin ameliorates diet-induced obesity and promotes browning of white adipose tissue by upregulating IRF4/PGC-1α
Source: Nutr Metab (Lond). 2022 Jun 11;19:38. doi: 10.1186/s12986-022-00672-6 (PMC9188085; doi:10.1186/s12986-022-00672-6)

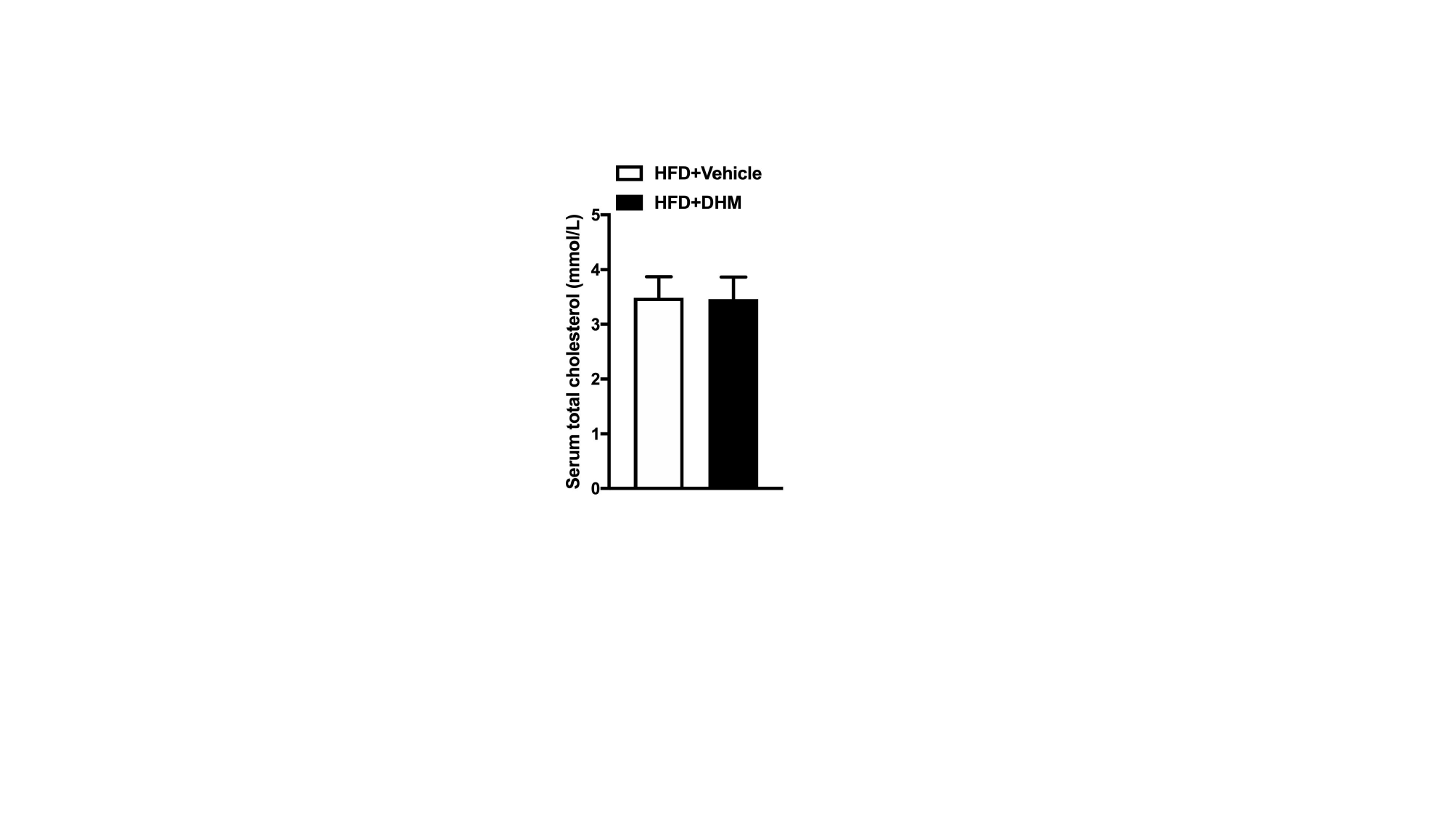

Supplement: Supplementary file 2 — Additional file 2. Fig. S1. DHM had no effect on serum TC. Serum triglyceride level (n=5). Data are presented as the mean ± SEM. *P < 0.05, **P < 0.01, and ***P <0.001. [file 12986_2022_672_MOESM2_ESM.tif]

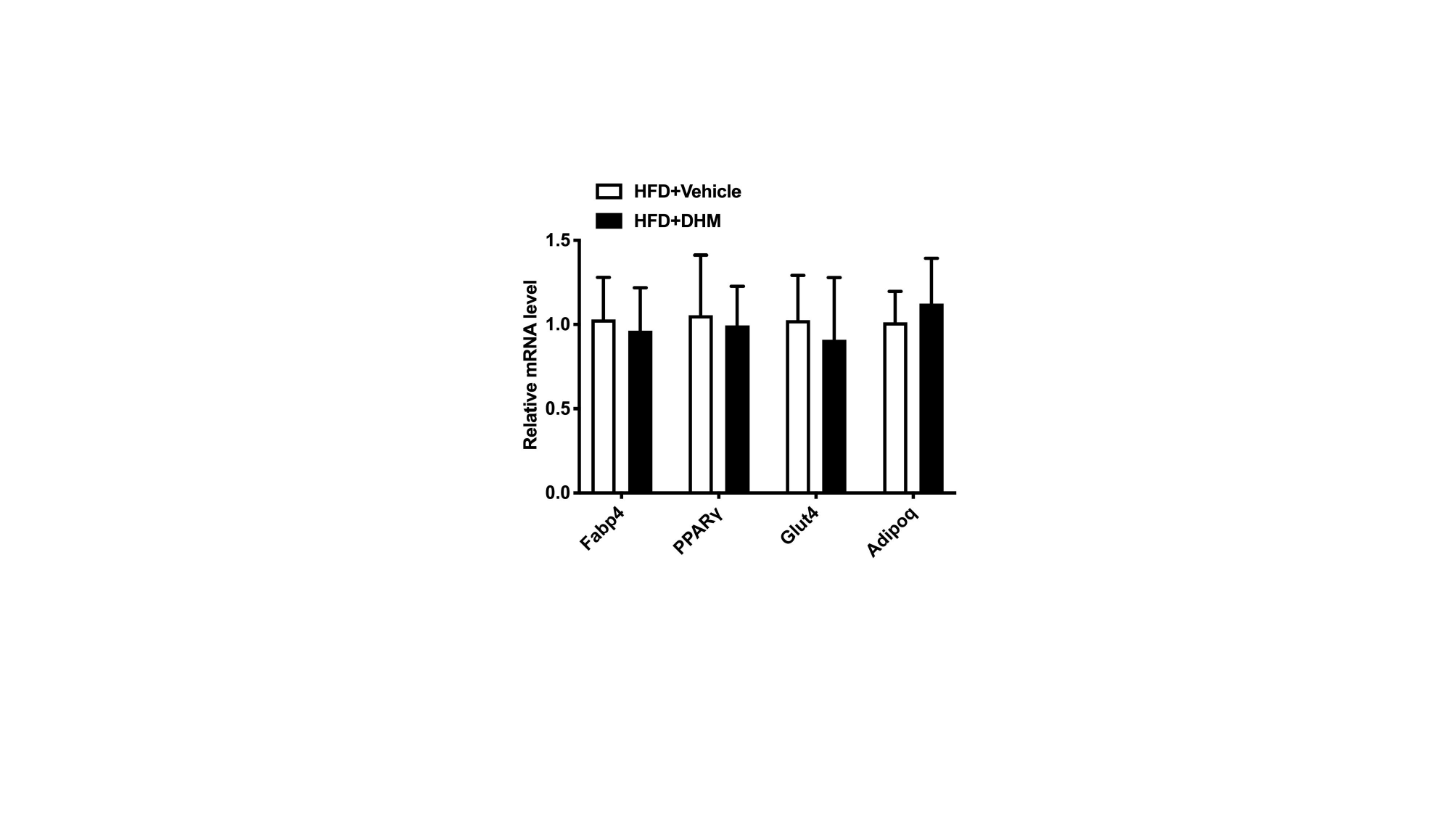

Supplement: Supplementary file 3 — Additional file 3. Fig. S2. DHM did not inhibit lipid synthesis of iWAT. RNA expression profiles of the adipocyte differentiation and lipogenesis related genes in iWAT. Data are presented as the mean ± SEM. *P < 0.05, **P < 0.01, and ***P <0.001. [file 12986_2022_672_MOESM3_ESM.tif]

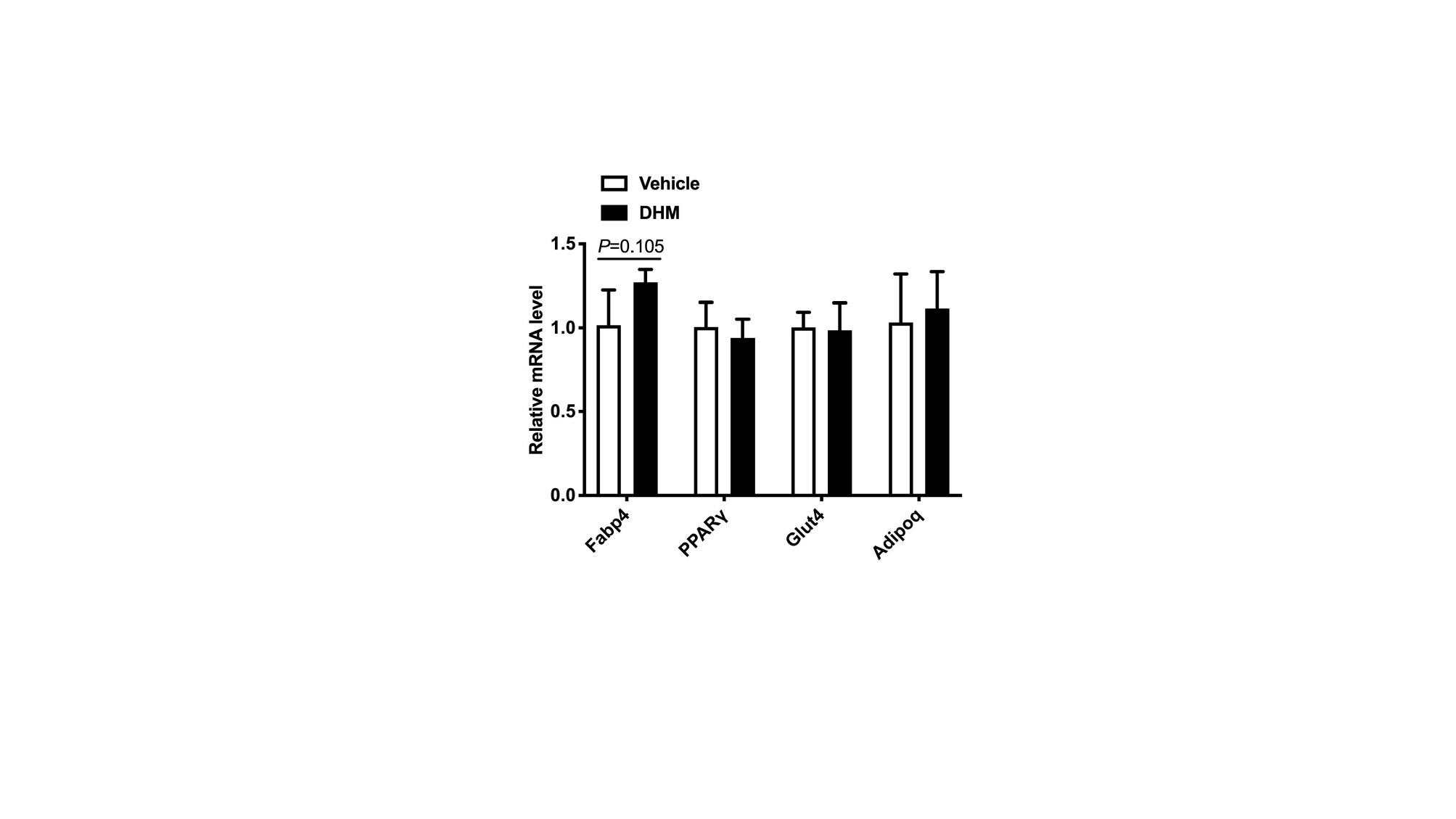

Supplement: Supplementary file 4 — Additional file 4. Fig. S3. DHM had no effect on lipid synthesis of primary adipocytes. RNA expression profiles of the adipocyte differentiation and lipogenesis genes in primary adipocytes. Data are presented as the mean ± SEM. *P < 0.05, **P < 0.01, and ***P <0.001. [file 12986_2022_672_MOESM4_ESM.tif]
